# Hyaluronic acid/diminazene aceturate combination ameliorates osteoarthritic anomalies in a rodent model: a role of the ACE2/Ang1-7/MasR axis

**DOI:** 10.1007/s10787-023-01335-5

**Published:** 2023-09-19

**Authors:** Yasser H. Habib, Eman Sheta, Mahmoud Khattab, Mennatallah A. Gowayed

**Affiliations:** 1 Department of Pharmacology and Toxicology, Faculty of Pharmacy, Cairo University, Cairo, Egypt; 2 Department of Pathology, Faculty of Medicine, Alexandria University, Alexandria, Egypt; 3 Department of Pharmacology and Therapeutics, Faculty of Pharmacy, Pharos University in Alexandria, Canal El-Mahmoudia Str., Smouha Alexandria, Egypt

**Keywords:** Osteoarthritis, Oxidative stress, Inflammatory mediators, Renin-angiotensin system, Hyaluronic acid, Diminazene aceturate

## Abstract

The implication of the tissue-localized renin-angiotensin system (RAS) in the pathogenesis of osteoarthritis (OA) has been documented in the last decades. A combination of intraarticular (IA) corticosteroid and hyaluronic acid (HYAL) is approved for pain relief in patients with mild to moderate OA. Combining HYAL with an activator of angiotensin-converting enzyme 2, diminazen aceturate (DIZE), was evaluated in this study for its therapeutic potential. Monosodium iodoacetate was used to induce OA. The effects of daily administration of DIZE versus once-per-week IA injection of HYAL and a combination of both drugs for 21 days on OA deformities in rats’ knees were observed. Evaluation of motor activities, pain, and inflammatory response was done using rotarod, knee bend, and knee swelling tests. RAS components, inflammatory biomarkers, and oxidative stress mediators were measured in the knee joint. X-ray radiological examination and histopathological investigations were used to assess joint degeneration and regeneration. Levels of both inflammatory and oxidative markers in knee joint homogenate of OA rats rose, and these increments were mostly improved by the three therapies with a more prominent effect of the drug combination, an effect that was also reflected in the behavioral tests. RAS markers have shown better responsiveness to the combination therapy over both drugs individually, showing a pronounced increase in the angiotensin 1–7 amount. Both radiological and histopathology investigations came to confirm the biochemical results, nominating a combination of HYAL and DIZE as a possible therapeutic option for OA.

## Introduction

Osteoarthritis (OA) is a chronic heterogeneous disorder in which the affected patients suffer from synovial joint failure. Many pathological changes occur in OA joints such as progressive loss and destruction of articular cartilage, degeneration of ligaments, subchondral bone thickening, boney spares osteophytes formation on the knee, synovium inflammation with variable degrees, and hypertrophy of the joint capsule (Felson 2006).

In a healthy joint, the cartilage is in a state of balance between the synthesis of the matrix and its degradation. Healthy cartilage consists of an intertwined complex of collaged fibers with proteoglycan, in which hyaluronan is the backbone of each proteoglycan molecule. This complete structure of the cartilage provides the joint its properties (Yamanishi et al. 2002; Moreland 2003). On the contrary, in OA joints, the degradative activities outweigh the synthetic activity. Inflammatory mediators such as interleukin-1 (IL-1) and tumor necrosis factor (TNF-α) are released in the joint disrupting this balance (Pelletier et al. 2001; Moreland 2003). Elevated oxidative molecules like NOX-4 and MMP-13 degrade extracellular components, cellular membranes, and nucleic acids leading to impaired biological activity and changes in protein structure. Consequently, the accumulation of damaged proteins in the tissue results in the apoptosis of chondrocytes and damage to the cartilage of the knee joints (Morel et al. 2015; Han et al. 2022).

In last years, the renin-angiotensin system (RAS)-related components have played an important role in the occurrence of OA. The RAS is formed out of two axes, the classical one, formed by angiotensin-converting enzyme (ACE), angiotensin (Ang) II and AT1 receptor (AT1R), and the counter-regulatory one, composed of Ang 1–7 and the Mas receptor (MasR). The two axes work counteractively, in which the classical one activates pro-inflammatory mediators and oxidative stress, and the Ang 1–7 exerts anti-inflammatory action, decreasing cytokine release, leukocyte attraction, tissue damage, and fibrosis (Moreira et al. 2021). In OA, there is an upper hand to the classical axes over the counterregulatory one, as Ang II increases the expression of NADPH oxidase which is associated with an increase in the ROS production (Hagiwara et al. 1998; Santos et al. 2003; Paul et al. 2006), confirming the role of RAS in the etiology of OA (Moreira et al. 2021).

Hyaluronic acid (HYAL) is found normally in many tissues such as the dermis, thoracic lymph, and synovial fluid, but the highest amount of HYAL is found in the extracellular matrix of the soft connective tissues (Iannitti et al. 2011). Normally, it binds to specific receptors found in different cells such as the intracellular adhesion molecule-1 (ICAM-1) and the receptor for hyaluronate-mediated motility (RHAMM), this triggers many intracellular signal events which help the body to have normal functional activities (Hodge-Dufour et al. 1997; Cao et al. 2005).

In OA, levels of HYAL are reduced compared to healthy joints, and administration of exogenous HYAL intraarticularly (IA) restores the viscoelastic properties of the joints by several concurrent mechanisms. HYAL has been shown to reduce the levels of oxidative stress and inflammatory mediators such as MMP-13, prostaglandin E2, IL-6, IL-1β, and TNF-α, besides its role in enhancing chondrocytes synthesis and proteoglycans, promoting cartilage regeneration and preventing its degradation (Altman et al. 2015). Moreover, it has a mechanical effect as it is shown to lubricate the joint capsule and prevent degradation through reducing the friction, side by side with its analgesic effect as many studies showed that it has a pain-relieving effect through acting on HYAL receptors at the free nerve ending within the knee joint (Gotoh et al. 1993; de la Peña et al. 2002; Moreland 2003).

Clinically, a combination of IA corticosteroid and HYAL is frequently used to improve the therapeutic profile of OA patients, and the action of both therapies in a combined form can cause a superior effect on symptomatic pain relief when compared with each drug individually (Smith et al. 2019). However, IA corticosteroid injections have been shown to provide short-term symptomatic relief with no long-term efficacy. Besides causing a transient elevation in the blood sugar level, which might be a concern for diabetic patients (Choueiri et al. 2021; Stone et al. 2021). Moreover, regular IA corticosteroid injections for 3 months have proven greater loss of cartilage, worse joint space narrowing, and increased risk of joint replacement (Ayub et al. 2021).

Diminazene aceturate (DIZE) is an antiparasitic–antitrypanosomal drug. Researchers found that DIZE has antioxidant and anti-inflammatory effects decreasing the levels of pro-inflammatory cytokines such as IL-1β and TNF-α, in addition, to downregulating the activity of NF-κB, p65, and p38 MAPK. It was found that DIZE increases endogenous ACE2 activity in an endotoxin-induced uveitis rat model and in ethanol and acetic acid mice models of gastric mucosal damage. This in turn stimulated the ACE II/Ang 1–7/ MasR cascade, resulting in decreased inflammation (Zheng et al. 2015; Souza et al. 2016). Being able to affect RAS-related components, besides its anti-inflammatory potential, nominates DIZE as a potential immunomodulator in OA.

In this study, the effects of both HYAL and DIZE on oxidative stress molecules, inflammatory mediators, and also different components of the RAS such as ACE1, ACE2, Ang 1–7, and MasR were evaluated in the OA rodent model. Both drugs were tested individually, as well as in combination for their potential to improve the OA-induced anomalies.

## Materials and methods

### Animals

Adult male Sprague–Dawley rats (170–200 g) were bought from the animal house facility of the Faculty of Pharmacy, Pharos University in Alexandria, Alexandria, Egypt. Five rats were kept under observation in each cage in a standard environmental condition (23–25 °C, 12 h light/dark cycle) prior to the study with free access to food and water. All animal manipulations in the experiment were performed according to the instruction of the Egyptian guide for the care and use of laboratory animals and in accordance with ARRIVE guidelines and the “National Research Council’s Guide for the Care and Use of Laboratory Animals”. The study experiments were approved by the “Research Ethical Committee” of the Faculty of Pharmacy, Cairo University, Cairo, Egypt (Approval No.: PT 3170).

### Drugs and chemicals

Monosodium iodoacetate and diminazen aceturate were obtained from Sigma-Aldrich, St. Louis, MO, USA. Hyaluronic acid (FIDIA PHARMA MIDDLE EAST, Dubai, United Arab Emirates) and ketamine/xylazine (Biochemie GmbH, Vienna, Austria) were obtained from commercial suppliers. Drugs were dissolved in saline before use.

### Induction of knee osteoarthritis (OA)

Rats received a single intraarticular (IA) injection of monosodium iodoacetate (MIA) at a dose of 3 mg/50 μL in sterile saline, into a disinfected and shaved right knee joint (Gupte and St Mart 2013; Hanafy and El-Ganainy 2020). Rats were subject to anesthesia (ketamine/xylazine; 50/5 mg/kg) before the IA injection (Ferreira-Gomes et al. 2008; Ziaei et al. 2016). All animals were left for 20 days to permit the progression of the knee OA (Hanafy and El-Ganainy 2020).

### Experimental design

**Fig. 1:**
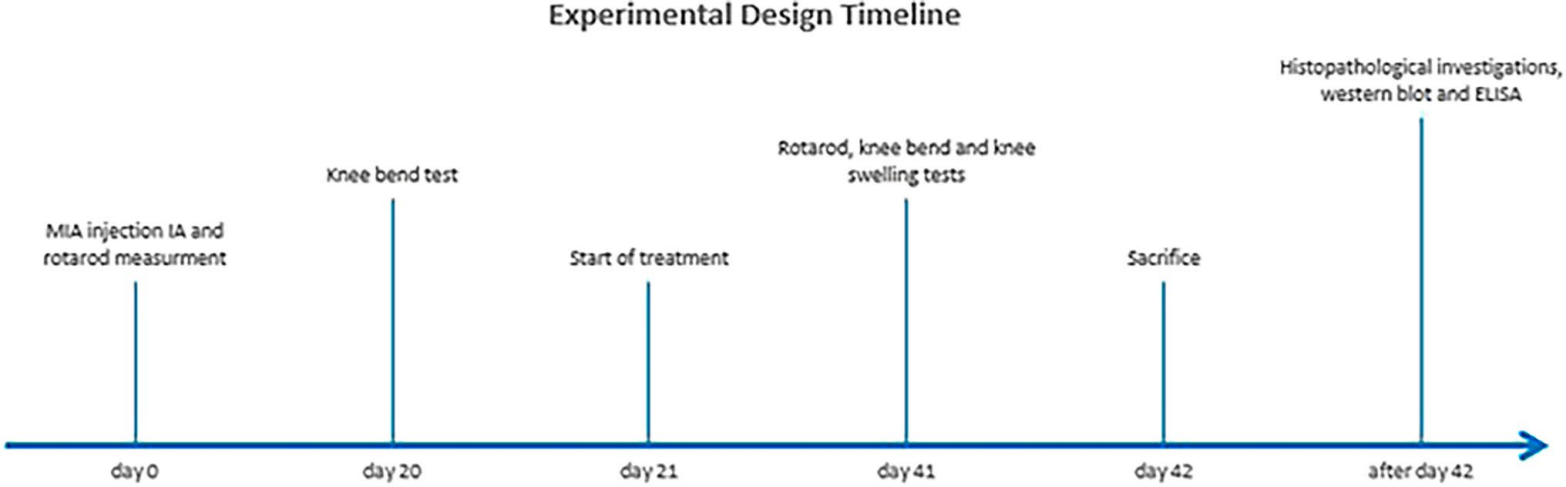
The experimental design timeline

Rats administered MIA were randomly distributed into four groups of ten rats each (1) OA rats: received IA saline (0.1 mL) once per week, (2) HYAL rats: OA rats received IA hyaluronic acid (50 μL of 10 mg/mL) once weekly (Ikeuchi et al. 2015; Salamanna et al. 2019), (3) DIZE rats: OA rats treated with DIZE (15 mg/kg/day) intraperitoneal (Awwad et al. 2020), and (4) HYAL/DIZE rats: OA rats received a combination of both treatments. In addition, a fifth group of normal healthy non-OA rats (CN) received saline intraarticularly (0.1 mL) once per week. Drugs were administered starting from day 21 after MIA administration for 21 days. Behavioral tests were conducted 24 h after the last drug administration (day 42). They included evaluation of motor functions, inflammation, and pain using rotarod, knee swelling, and knee bent tests, respectively. Finally, rats were euthanized by an overdose of phenobarbital 200 mg/kg i.p. The injected knee joints were separated and stored at − 80 °C for further biochemical analysis. From each group, four injected knee joints were fixed in formalin (10%) for histopathological examination. Figure 1 shows the timeline of the experiment.

### Behavioral tests

#### Rotarod test

A rotating rod instrument was used to assess the motor coordination and balance of rats enrolled in the experiment. One week before induction, rats were trained to stay on the rotarod for 5 min (20 rpm) to produce forced motor activity. The training was applied for three consecutive days, followed by the test on day zero before induction. The test was repeated before the sacrifice (day 41), where the rats were allowed to move on the rotating rod until falling (20 rpm). Three falling latencies were recorded on the test day with 15 min rest between measurements, and the average was calculated for statistical analysis (Piel et al. 2014; Hanafy and El-Ganainy 2020). The % of the change in falling latency from day zero was calculated.

#### Knee bend test

A knee bend test was performed to evaluate OA-related knee joint pain. The right knee joint was exposed to five alternate extensions and flexions. The animal struggle and/or squeaks were considered a positive reaction, counted, and scored: 0—no reaction to joint movement, 0.5—reaction to maximal extension or flexion, 1—reaction to moderate extension or flexion, or vocalization to maximal extension or flexion, 2—vocalization to moderate extension or flexion. The test was performed on days 20 and 41 (before and after treatment). The mean total score for each group was calculated as an indication of the grade of the nociception (Hanafy and El-Ganainy 2020).

#### Knee joint swelling

A knee joint swelling test was performed to evaluate the OA-related joint swelling and inflammation. At the end of the experiment (day 41), rats were sacrificed, the skin around the knee was cut open and the knee diameter was determined by a vernier caliper as an indicator of inflammation and edema. (Hanafy and El-Ganainy 2020).

### X-ray radiography

On day 41 before sacrifice, rats were anesthetized using ketamine/xylazine (50/5 mg/kg) and injected right knee joints were examined using a digital vet X-ray (Poskom Co., Ltd., Korea). Morphometric changes of the tibia and the femur (between the epiphysial growth plates) were assessed, in addition to the articular cartilage. A specialized radiologist who was blinded to the experimental design assessed the resulting knee joint images. Kellgren–Lawrence grading scale from 0 to 4 was used. A score of 0 for images that show no sign of osteoarthritis; a score of 1 for images that show suspected osteophyte formation or joint space narrowing; a score of 2 for images that show certain osteophyte development and probable joint space narrowing; a score of 3 for images that show various osteophytes, certain sclerosis and joint space narrowing, as well as probable bone deformity; a score of 4 for images that show end-stage, marked by severe sclerosis, joint space narrowing (sometimes bone-on-bone contact), and large osteophytes (Thomas et al. 2020).

### Knee tissue analysis

After sacrifice, articular cartilage of the injected right knee was removed under anesthesia using pentobarbital overdose; 200 mg/kg (Laferriere and Pang 2020). The removed cartilage was cut into thin sections and divided into two parts. One part was used for ELISA measurement of the RAS biomarkers: ACE1 (Cat. No. MBS733102), ACE2 (Cat. No. MBS764117), Ang 1–7 (Cat. No. MBS2604372) and Ang II (Cat. No. MBS705139) content. The Inflammatory biomarker transforming growth factor beta (TGF-β1, Cat. No. MBS824788), as well as the oxidative stress biomarker NOX-4 (Cat. No. MBS2503069) content were also determined. All ELISA kits were purchased from myBioSource, CA, USA and determined in knee tissue homogenates according to the manufacturer’s instructions.

The second part of the cartilage was homogenized in the Laemmli sample buffer (SDS 4%, 2-mercaptoethanol 10%, glycerol 20%, bromophenol blue 0.004% and 0.125 M Tris HCl) for western blot analysis. The pH was adjusted to 6.8. Preceding mixtures were boiled (95 °C) for 5 min to confirm the denaturation of protein before the loading on gel electrophoresis. Bradford Protein Assay Kit (SK3041, Bio basic nc., Markham Ontario L3R 8T4 Canada) was used for quantitative protein analysis in the supernatant. The electrophoresis technique was used to resolve the protein samples (20–30 μg) on 8–10% sodium dodecyl sulfate–polyacrylamide gel (SDS-PAGE) and then electroblotted onto polyvinylidene fluoride (PVDF) membranes. 3% bovine serum albumin tris-buffered saline with Tween 20 (TBST) buffer were used to attain the membrane blocking by 1-h incubation at room temperature. An overnight incubation (4 °C) of membranes with primary antibodies was followed. Anti-MasR (Cat. No. 3707, cell signaling technology, MA, USA) as a RAS biomarker, anti-TNF-α (Cat. No. G-1: sc-390453, Santa Cruz biotechnology, CA, USA) as inflammatory marker, and anti-MMP-13 (Cat. No. C-3: sc-515284, Santa Cruz biotechnology, CA, USA) as oxidative stress biomarker were used. Subsequently, incubation with HRP-conjugated secondary antibodies was allowed to progress for 1 h at room temperature. The bands were visualized using a chemiluminescence detection system. ChemiDoc MP imager (Media Cybernetics, Bio-Rad's, INC, Canada) was used to measure the band strength of the test proteins against the housekeeping protein β-actin as control (anti-β-actin antibody, Cat. No. MA5-15739, Thermofisher, MA, USA).

### Histopathological examination

For histopathologic assessment of pathologic changes in the knee joint of different studied groups, the right knee joint was excised and bisected longitudinally after decalcification. The bisected joints were then processed into paraffin blocks. Serial sectioning by a rotatory manual microtome was done (5 microns thick) and sections were mounted on glass slides. One slide was stained by hematoxylin and eosin while the other was stained by safranin O fast green (SOFG) stain. Both slides were examined to assess the osteoarthritis-related changes. The OARSI histological score was used to assess the articular cartilage (Udo et al. 2016). Meanwhile, the synovium was evaluated for the presence of fibrosis, inflammation, and hyperplasia in the infrapatellar fat pad. Each of the synovitis and fibrosis were scored out of 3 then both scores were summed into a final score out of 6 (Pritzker et al. 2006). A digital camera was used to capture different images of articular cartilage and the thickness from surface to subchondral bone was measured in microns by Leica application suite version 4.12 software.

### Statistical analysis

Values are expressed as mean ± S.D. (*n* = 10). One-way analysis of variance (ANOVA) followed by the Tukey post hoc test was used to analyze the data. For nonparametric data, results were analyzed by Kruskal–Wallis Test, followed by Dunn's post hoc test. Regression analysis was performed to examine the synergistic effect of the drug combination. The significant limit for all comparisons was considered at *p* < 0.05. Analysis and presentation of data were performed by the GraphPad Prism 5.0 software (GraphPad Prism Inc., La Jolla, CA, USA).

## Results

### Effect of HYAL, DIZE, and their combination on osteoarthritis-associated behavioral changes

#### Effect on motor function

**Fig. 2:**
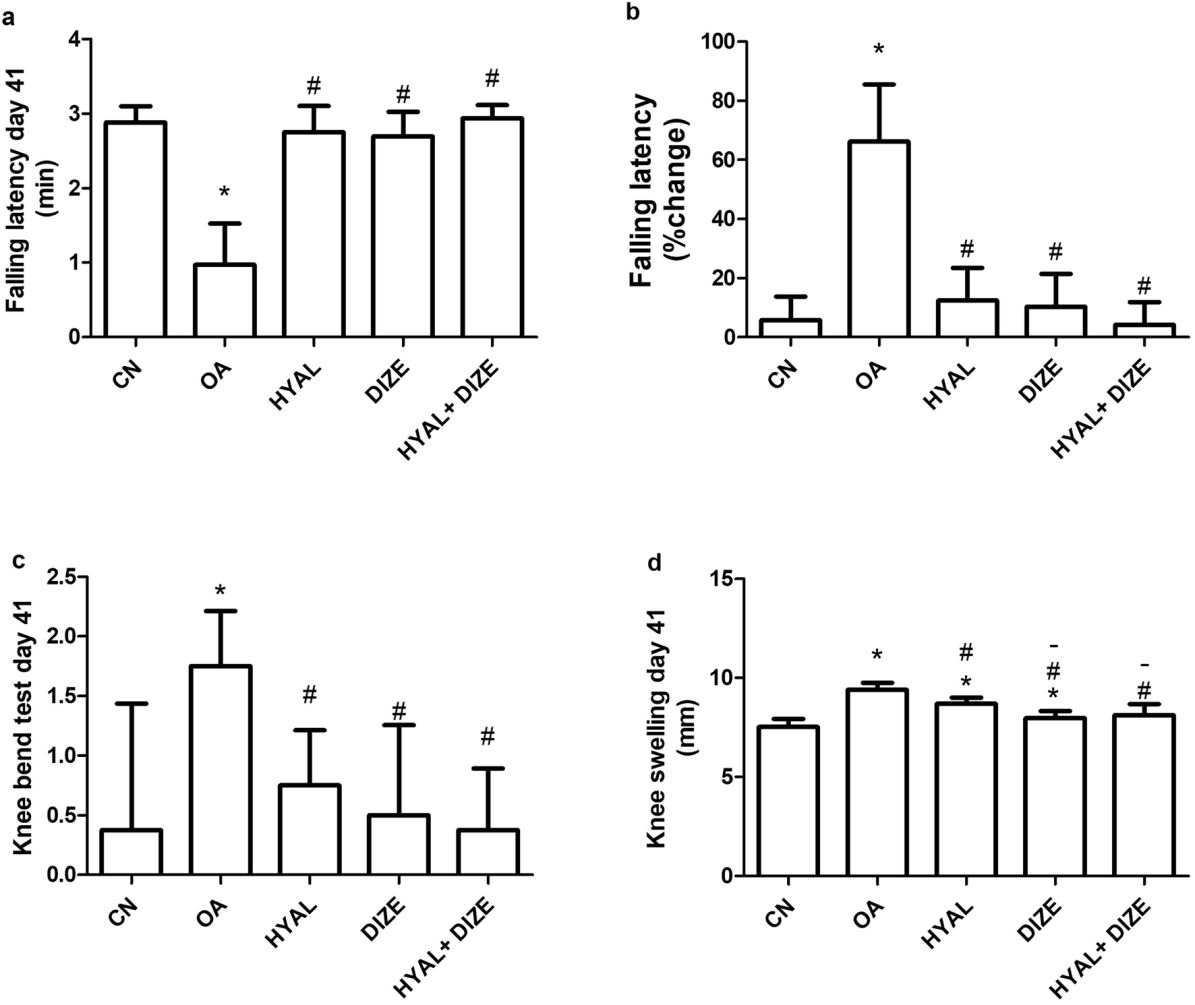
Evaluation of osteoarthritis-associated behavioral changes. **a**, **b** Rotarod test showing the falling latency at the end of the treatment period on day 41 in (**a**) and % change from day zero in (**b**), **c** knee bend test and **d** knee swelling test on day 41. Data are shown as means ± SD (*n* = 10). One-way ANOVA was used for comparing the different groups, followed by the Tukey post hoc test. In the knee bend test data were analyzed using Kruskal–Wallis test, followed by Dunn's post-hoc-test. Data are compared to (*) CN, (#) OA and (–) HYAL at *p* < 0.05. CN, control; OA, osteoarthritis; HYAL, hyaluronic acid, DIZE, diminazene

The Rotarod test allows the assessment of motor activity including motor function and coordination. Variations in rotarod measurements resulted from a single intraarticular injection of MIA at a dose of 3 mg/kg in treated or untreated adult male rats as shown in Fig. 2a, b. OA rats showed a significant reduction in rotarod performance on day 41 of the study compared to the negative control group. The weekly administration of HYAL (50 μL of 10 mg/mL), diminazene aceturate (15 mg/kg/day) and combination therapy of both drugs caused a significant equipotent enhancement of the rotarod performance compared to the OA untreated group (66.19% of change for OA vs 12.5% for HYAL, 10.30% for DIZE and 4.167% for HYAL + DIZE).

#### Effect on OA-associated knee pain

The knee bend test helps to assess knee pain. Before treatment (day 20) all rats injected with MIA presented significant and remarkable increments in vocalization and pain upon maximal extension or flexion (Table 1). All three treated groups were able to reverse the signs of pain at the end of the treatment period on day 41 (mean score 0.75 HYAL, 0.50 DIZE and 0.375 HYAL + DIZE), Fig. 2c.

**Table 1:** Results of the knee bend test score before and after treatment

	CN	OA	HYAL	DIZE	HYAL + DIZE
Day 20	0.000 ± 0.000	1.250^*^ ± 0.2500	1.250^*^ ± 0.2500	1.500^*^ ± 0.1830	1.500^*^ ± 0.1890
Day 41	0.3750 ± 0.2631	1.750* ± 0.1637	0.7500^#^ ± 0.1637	0.5000^#^ ± 0.2673	0.3750^#^ ± 0.1830

Comparisons among groups were analyzed using Kruskal–Wallis Test followed by Dunn's post hoc test. Data are compared at *p* < 0.05 with CN (*), OA (#). Values are presented as means ± SEM (*n* = 10). Control (CN), osteoarthritis (OA), hyaluronic acid (HYAL) and diminazene aceturate (DIZE)

#### Effect on knee swelling

As shown in Fig. 2d, induction of OA resulted in a significant increase in knee swelling, showing a 25% increase from control rats on day 41. All treated groups have shown a significant decrease in knee swelling diameter, however, treatment with DIZE alone or in combination showed the best effect (For DIZE: *p* < 0.001 vs OA and *p* < 0.01 vs HYAL. For HYAL + DIZE: *p* < 0.001 vs OA and *p* < 0.05 vs HYAL).

### Effect of treatment on osteoarthritic X-ray radiography

**Fig. 3:**
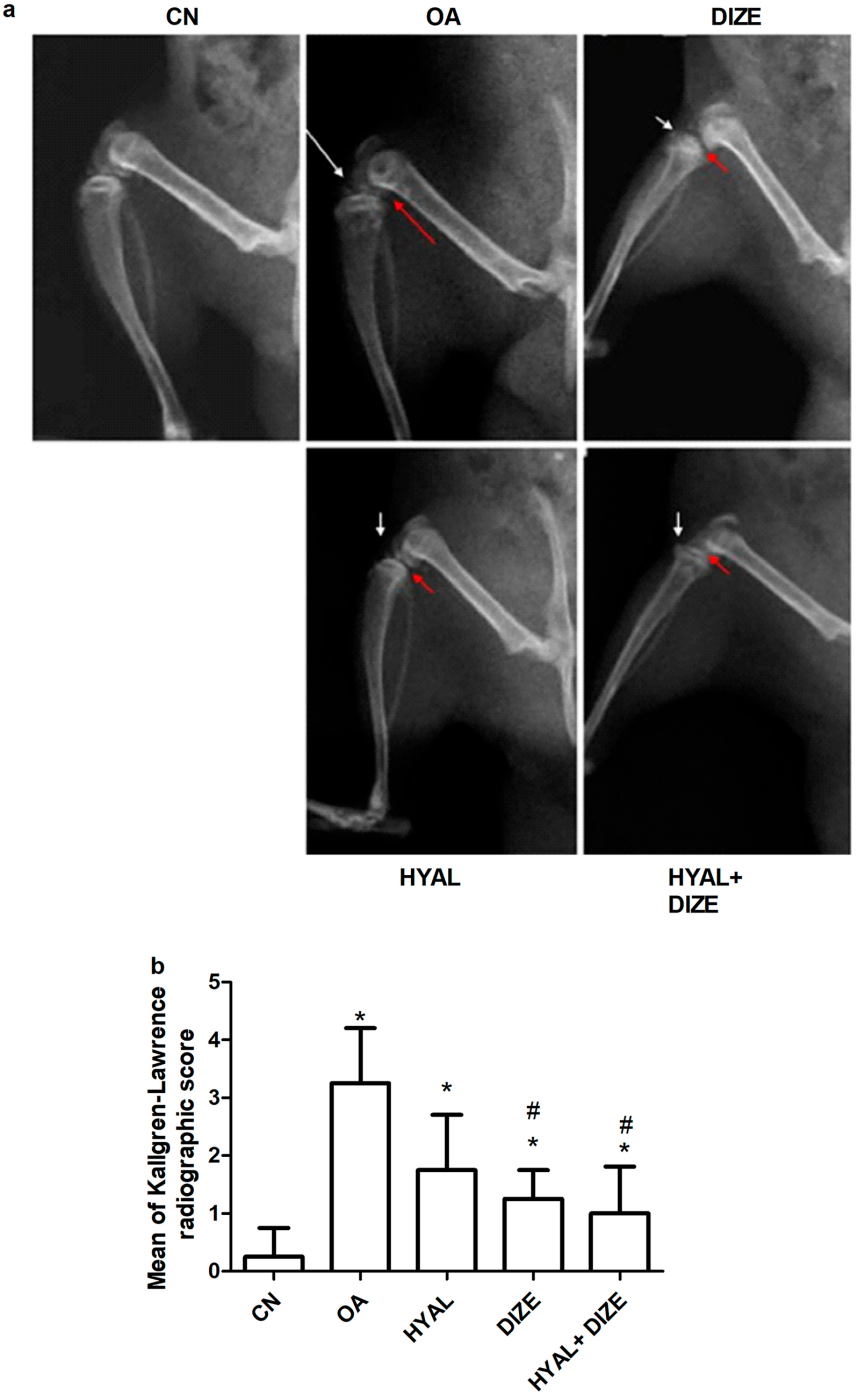
Evaluation of the osteoarthritis-associated radiological changes. Assessment of the right knee joint is shown in (**a**) as representative lateral view radiographs. The mean of the Kellgren–Lawrence radiographic score is shown in (**b**). White arrows indicate osteophytes and red arrows indicate joint space narrowing. Data are shown as means ± SD (*n* = 4). One-way ANOVA was used for comparing the different groups, followed by the Tukey post hoc test. Data are compared to (*) CN and (#) OA at *p* < 0.05. CN, control; OA, osteoarthritis; HYAL, hyaluronic acid, DIZE, diminazene

Radiographic imaging revealed a pronounced joint space narrowing of OA knees (Fig. 3a, b) showing a 75% increase in mean score compared to normal control rats. Moreover, the articular margins of OA knees were rough and sclerotic with profuse osteophytes in both ventrodorsal and lateral observations. Both the combination of HYAL with DIZE and DIZE monotherapy showed a more prominent effect than HYAL in improving the observed joint space narrowing (% decrease for DIZE: 50% vs OA and 13% vs HYAL. For HYAL + DIZE: 56.25% vs OA and 37.5% vs HYAL), Fig. 3b. All treated groups have shown a smooth surface with a significant decrease in osteophytes number.

### Effect of treatment on the knee tissue biochemical analysis

**Fig. 4:**
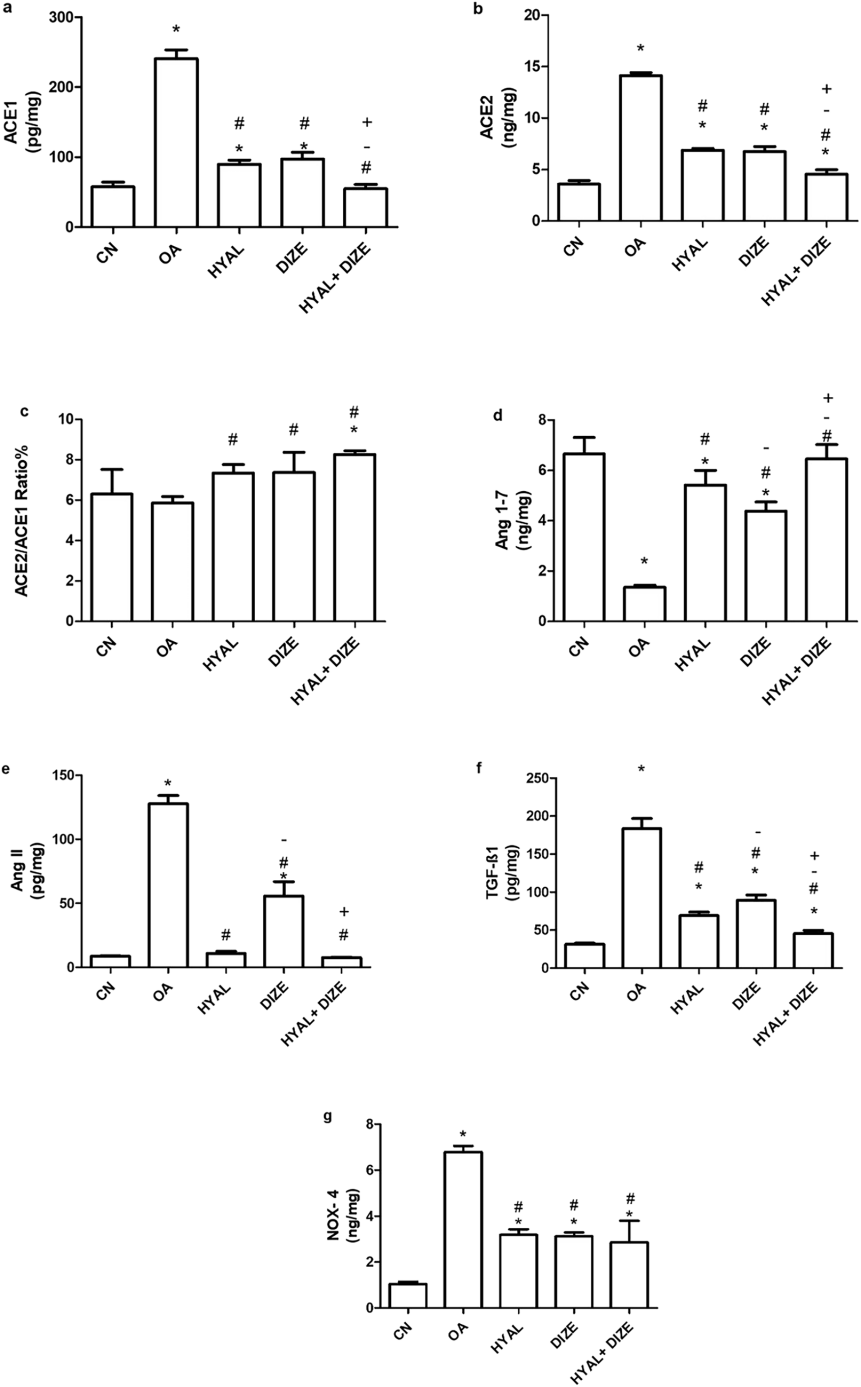
Effect of drug treatments on the knee tissue biochemical analysis. Knee tissue levels of **a** angiotensin-converting enzyme 1 (ACE1), **b** angiotensin-converting enzyme 2 (ACE2), **c** ACE2/ACE1ratio %, **d** angiotensin 1–7 (Ang 1–7), **e** Ang II, **f** transforming growth factor beta 1 (TGF-β1) and **g** NADPH oxidases 4 (NOX-4). Data are shown as means ± SD (*n* = 6). The tissue was analyzed using ELISA according to the manufacturer’s instructions. One-way ANOVA was used for comparing the different groups, followed by the Tukey post hoc test. Data are compared to (*) CN, (#) OA, (–) HYAL and (+) DIZE at *p* < 0.05. CN, control; OA, osteoarthritis; HYAL, hyaluronic acid, DIZE, diminazene

ELISA technique was used to measure knee tissue levels of ACE1, ACE2, Ang II, Ang 1–7, TGF-β1 and NOX-4 (Fig. 4). Results showed that compared with the healthy control group, the OA group presented a significant increment of ACE1, ACE2, Ang II, TGF-β1 and NOX-4 levels in knee tissues, while the Ang 1–7 level showed a significant reduction (Fig. 4d). Effects were restored significantly in all the three treated groups, where the combination therapy effectively caused a more significant change than the other two drugs in almost all studied parameters (*p* < 0.001 compared to OA), except NOX-4, where all therapies showed to be equally effective (Fig. 4g), *p* < 0.001. Calculation of the ACE2/ACE1 ratio percentages (Fig. 4c) demonstrated that all types of treatments (DIZE, HYAL, and DIZE + HYAL) increased this ratio above the OA value.

**Fig. 5:**
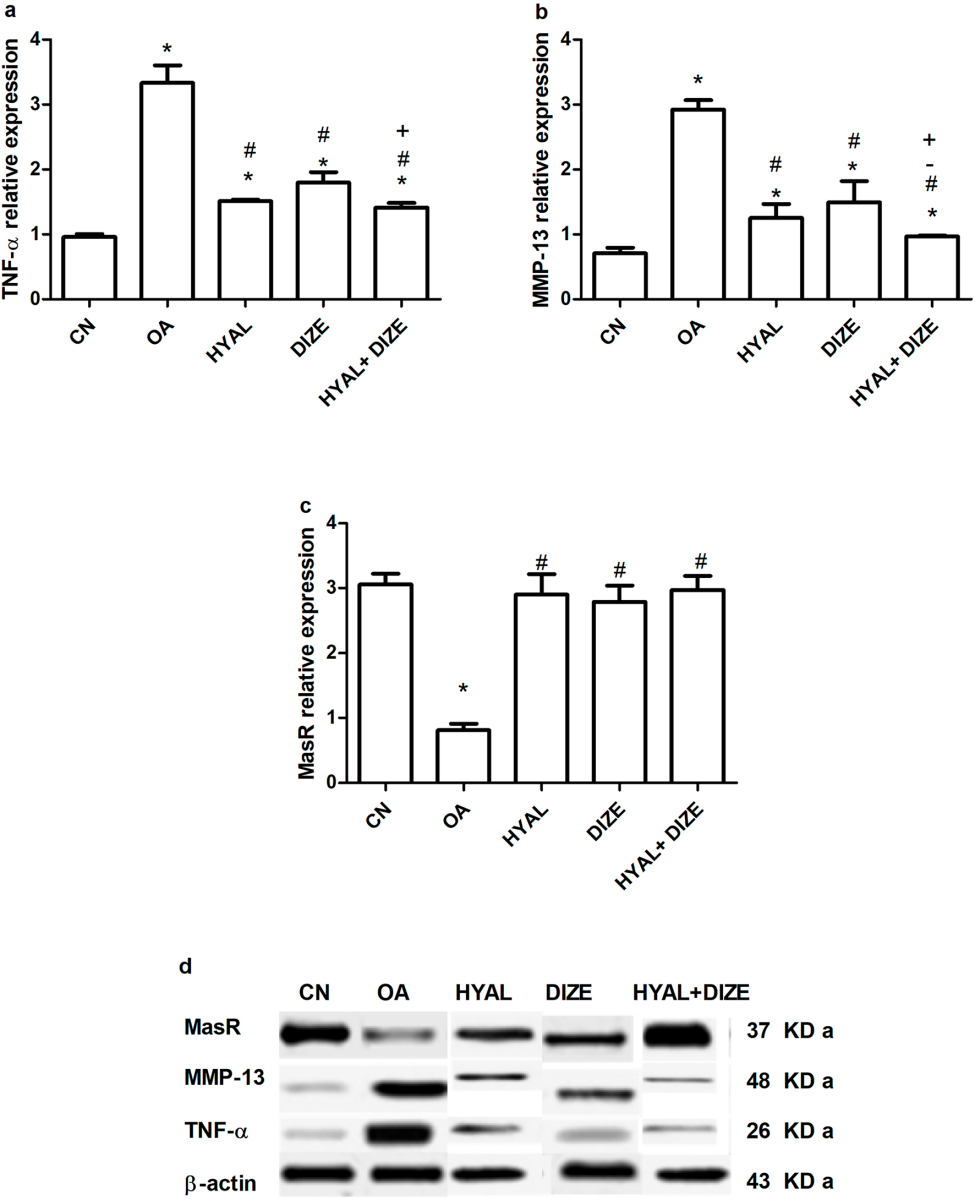
Effect of drug treatments on the knee tissue western blot analysis. Knee tissue levels of **a** tumor necrosis factor-alpha (TNF-α), **b** matrix metalloproteinases 13 (MMP-13), **c** mas receptor (MasR) and **d** representative western blot bands. Data are shown as means ± SD (*n* = 3). One-way ANOVA was used for comparing the different groups, followed by the Tukey post hoc test. Data are compared to (*) CN, (#) OA, (–) HYAL and (+) DIZE at *p* < 0.05*.* CN, control; OA, osteoarthritis; HYAL, hyaluronic acid, DIZE, diminazene

Western blot technique was used to measure knee tissue levels of the inflammatory TNF-α, MMP-13 as oxidative stress marker and MasR as part of the examined knee RAS. The TNF-α and MMP-13 levels have shown a significant elevation in induced OA rats (Fig. 5a, b), while the MasR knee level has shown a decrease (Fig. 5c) compared to the normal control group. All these previous anomalies were significantly reversed in all treated rats. Among the three treated groups, the group taken the combination therapy showed a more significant effect on the levels of TNF-α and MMP-13 (*p* < 0.001 vs OA, *p* < 0.01 vs HYAL,* p* < 0.05 and *p* < 0.001 vs DIZE, respectively). However, all three treated groups were equally effective in increasing the MasR level (*p* < 0.001 vs OA). Figure 5d shows representative western blot bands.

### Effect of treatment on knee histopathology

#### Pathologic assessment of synovial tissues

**Fig. 6:**
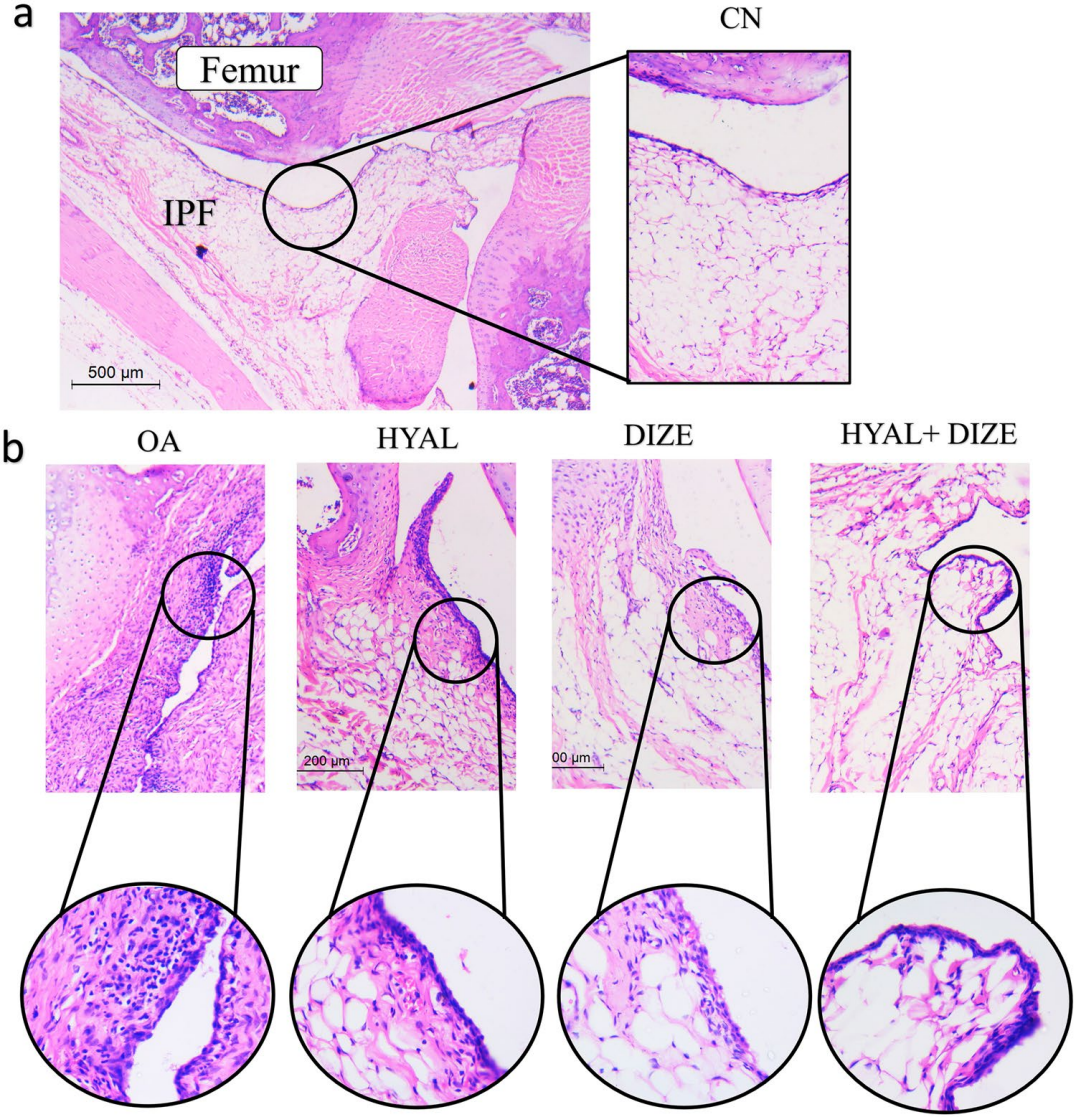
Synovial tissue assessment in H&E-stained sections of studied groups. **a** Low-power image of the knee joint to highlight different parts of the patellofemoral joint and the anatomical site of synovial tissues (× 40). Synovium in the CN group (× 100) showed fat cells with no synovial hyperplasia, inflammation, or fibrosis (score 0). **b** Synovium assessment in other groups: OA group showing wide dense fibrosis with totally absent fat. Inflammation is seen and scored as (5). The different treated groups showed variable decline of both synovitis and fibrosis scores (low power × 100, inset × 400)

In the CN group, the synovium is covered by one to two layers of flat synovial cells. No fibrosis or inflammatory infiltrate were noted. In opposite to the OA group, the synovial lining was thick and hyperplastic. More than two layers of synovial cells were seen. The sub-synovial tissues were fibrotic with inflammatory infiltrate noted. HYAL and DIZE-treated rats showed a decrease in both fibrosis and inflammation scores. The combined regimen of both treatments (HYAL + DIZE) markedly improved the synovial histology. Fibrosis was not seen, and inflammatory cells were only occasionally detected (Fig. 6).

#### Pathologic assessment of articular cartilage

**Fig. 7:**
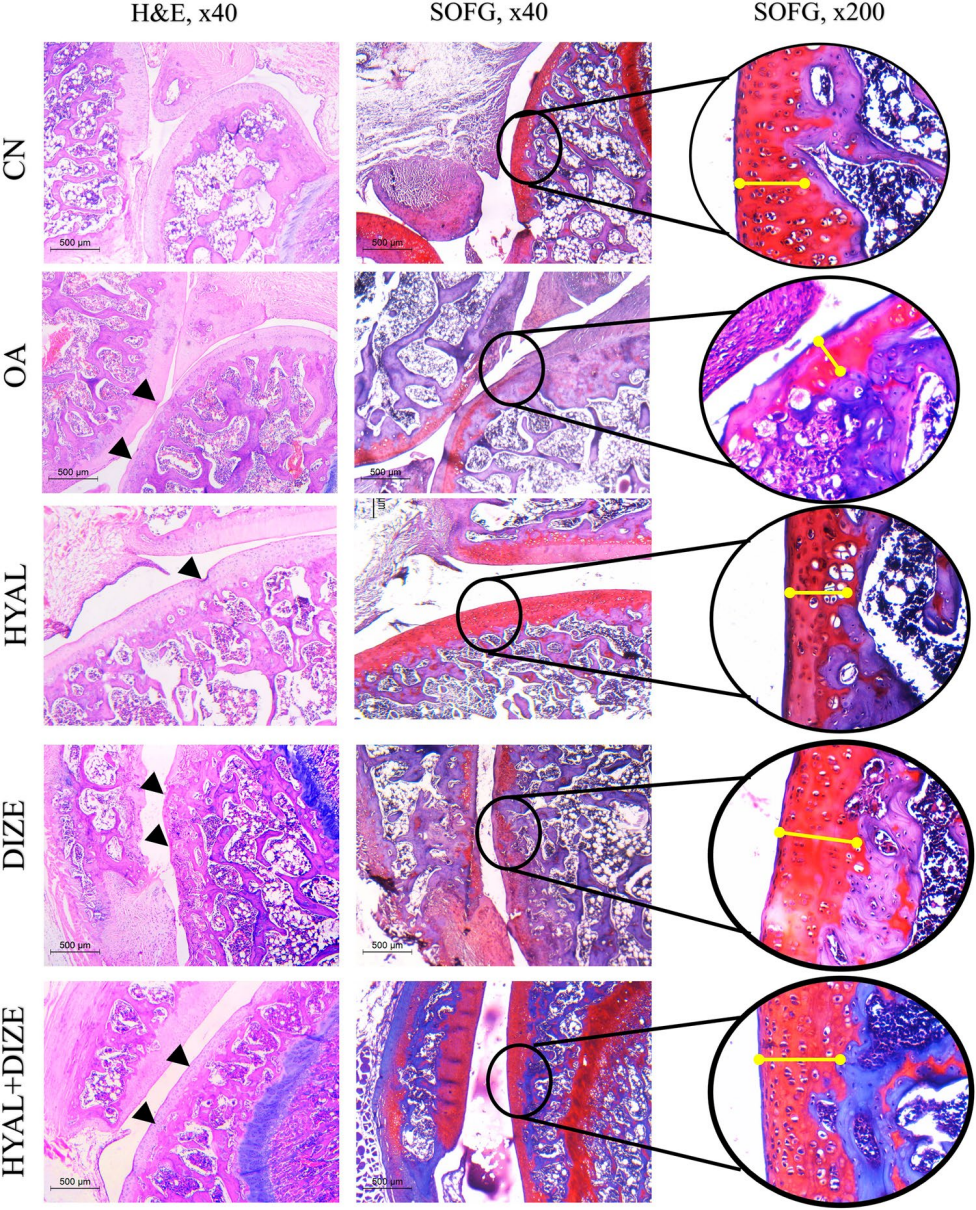
Assessment of articular cartilage in different studied groups in H&E and Safranin O fast green (SOFG) stained sections. The low power view of the CN group shows the normal architecture of the knee joint, SOFG stain shows high proteoglycan content (seen as red color). The OA group shows rough articular cartilage, decreased thickness and pale SOFG stain. In HYAL and DIZE-treated groups, mild roughness is still seen in articular cartilage (arrowheads). SOFG stained section shows increased proteoglycan in articular cartilage and increased thickness. In the HYAL + DIZE group, articular cartilage is smooth. Yellow lines represent articular thickness in different groups

**Fig. 8:**
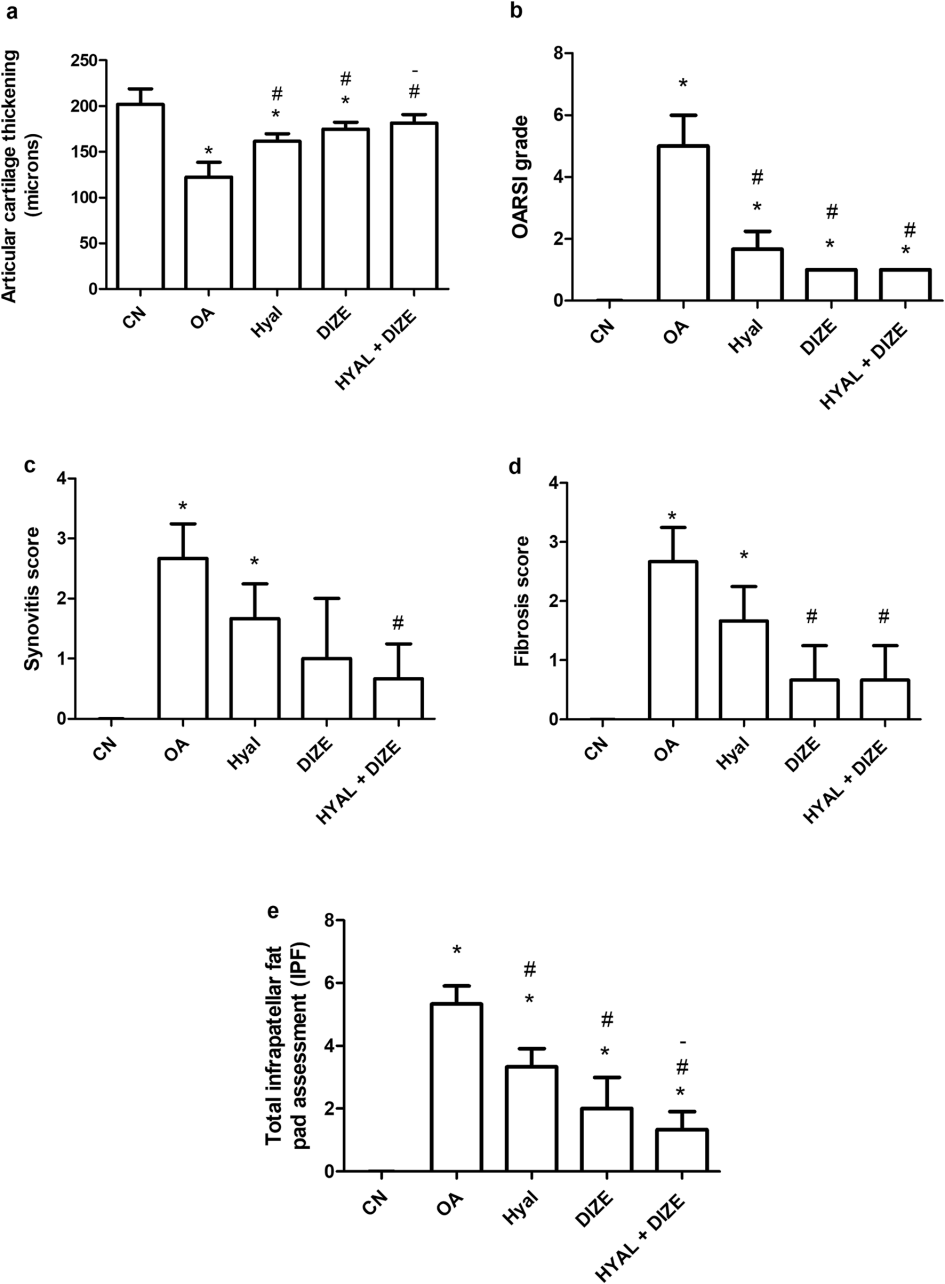
Effect of drug treatments on osteoarthritis-associated histopathology changes. The H&E and safranin O fast green stained sections show articular cartilage thickening (**a**), OARSI grade (**b**), synovitis (**c**), fibrosis (**d**) and total infrapatellar fat pad, IPF (**e**). The sum scores of synovitis and fibrosis make the total IPF score. Data are shown as means ± SD (*n* = 4). One-way ANOVA was used for comparing the articular cartilage thickening, followed by the Tukey post hoc test. Kruskal–Wallis test was used to analyze the OARSI grade and IPF scores, followed by Dunn's post-hoc test. Data are compared to (*) CN, (#) OA and (–) HYAL at *p* < 0.05*.* CN, control; OA, osteoarthritis; HYAL, hyaluronic acid, DIZE, diminazene

Sections of the knee joint in the CN group did not show any pathologic changes. The lower end femur, upper tibia, and patella were covered by a thick layer of articular cartilage. The SOFG stain was used to assess the proteoglycan content of the cartilaginous matrix (Fig. 7). It stained red indicating high proteoglycan content. The knee joints in the OA group showed a rough articular surface with abrasions. Weak SOFG staining is seen in many regions indicating proteoglycan depletion. The articular cartilage thickness markedly decreased in some areas. Chondrocytes showed a disorganized pattern with areas of hypocellularity. Nuclei were dark-stained and of different sizes. Hyaluronic acid treatment significantly alleviated the histopathologic changes in comparison to the OA group. The OARSI grade improved with an evident increase in the articular cartilage thickness (Fig. 8). The SOFG stained sections showed regaining the deep red color. Combined treatment slightly increased the articular cartilage thickness in comparison to each treatment when used alone. The chondrocytes regained their normal architecture in most areas.

### Regression analysis

**Fig. 9:**
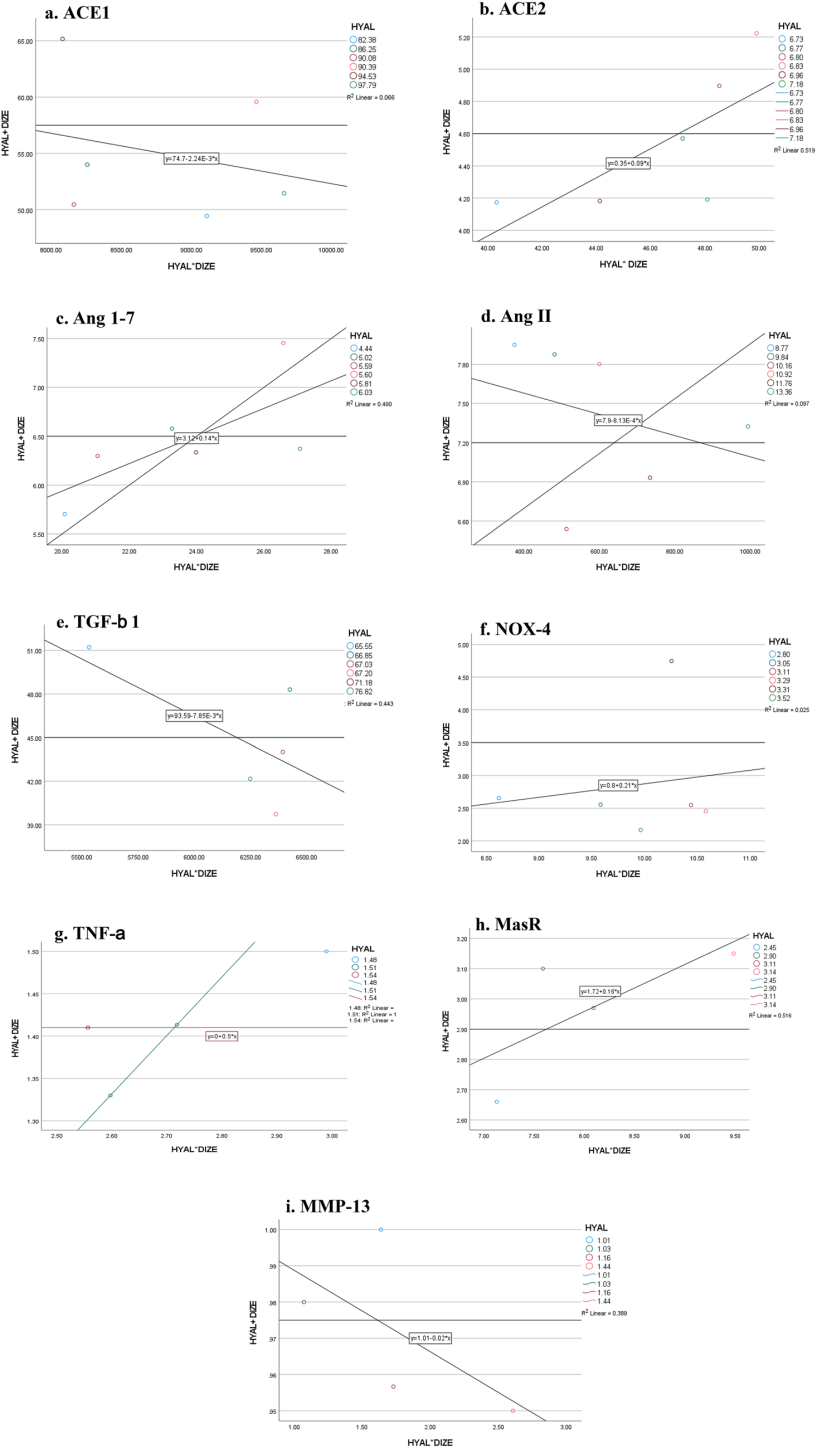
Regression analysis showing the possible synergistic effect of the combination therapy on the different parameters. **a** Angiotensin-converting enzyme 1 (ACE1), **b** angiotensin-converting enzyme 2 (ACE2), **c** angiotensin 1–7 (Ang 1–7), **d** Ang II, **e** transforming growth factor beta 1 (TGF-β1), **f** NADPH oxidases 4 (NOX-4), **g** tumor necrosis factor-alpha (TNF-α), **h** mas receptor (MasR) and **i** matrix metalloproteinases 13 (MMP-13). Data are compared at *p* < 0.05

Results of the regression analysis came to show a synergistic effect of HYAL + DIZE on the ACE2 (*p* = 0.038*, *R*^2^ = 88.7%) and TNF-α (*p* < 0.001*, *R*^2^ = 99.8%), while their effect on other parameters was not significant (Fig. 9).

## Discussion

The current study investigated the potential of hyaluronic acid with/without diminazene, the known ACE2 activator, to reprogram osteoarthritis-induced anomalies in a rat model. All the observed OA alterations were improved upon treatment, however, a combination of HYAL with DIZE has shown to be a promising therapeutic option, causing the most prominent improvement in almost all studied parameters, and confirmed by the histopathology and radiological examination. This combination has also shown a significant influence on both RAS-axis, highlighting its role in the immunomodulation of inflammatory OA.

Osteoarthritis induction caused fibrosis and inflammation of the synovial tissue, besides a rough surface of the articular cartilage, decreased chondrocytes amount with significant depletion in the proteoglycan content. Such histopathology appearance is commonly observed in monosodium iodoacetate (MIA)-induced OA models. Being a metabolic inhibitor, MIA breaks down the cellular aerobic glycolysis pathway, inducing the death of chondrocytes and morphological alterations of the cartilage (Lampropoulou-Adamidou et al. 2014), inhibiting normal knee function. This in turn caused a loss in motor activities and coordination, increased knee pain and swelling, and significant elevations in the inflammatory and oxidative stress biomarkers in joint tissue. Similarly, Manjhi et al. (2016) and Lee et al. (2018) have shown a decreased motor performance of OA rats in a papain-induced osteoarthritis rat model and collagen-induced arthritis rat model. Other studies confirmed the reliability of the knee bend and knee swelling tests in reflecting the degree of joint effusion as the main clinical feature of the OA (Ferreira-Gomes et al. 2008; Gupte and St Mart 2013; Ziaei et al. 2016).

The combination of diminazen and hyaluronic acid in the current study significantly improved motor performance, knee swelling and pain, observed radiologically as diminished soft tissue swelling, and improved synovial histology showing neither inflammatory cells nor fibrosis. The effect of HYAL on osteoarthritis has been proven previously (Migliore and Procopio 2015), where it has shown a gradual reduction of joint damage in an MIA-induced OA model (Halfaya et al. 2020). Additionally, the effect of DIZE on arthritis has been studied in a mouse model of rheumatoid arthritis decreasing the invasion of the synovial fibroblast into the cartilage, showing anti-inflammatory effects. This besides its ability to decrease the activity of synovial fibroblast obtained from RA patients (Neidhart et al. 2014; da Silva Oliveira and de Freitas 2015; Németh et al. 2022). Other studies also showed the anti-inflammatory effect of DIZE on different animal models such as the bovine mammary epithelial cells *in vitro* model and the myocardial infarction rat model (Qi et al. 2013; Jia et al. 2021).

The renin-angiotensin system has a significant role in controlling normal body balance, blood pressure, and homeostasis, having paracrine, autocrine, and intracrine functions on tissue and cellular levels (Wong 2016). Studies revealed that RAS and its components including ACE, Ang II, AT-1R and AT-2R have a major role in the etiology of OA and are involved in inflammation and chondrocyte anomalies (Wu et al. 2019). A clinical study showed that the imbalance of both RAS axes is associated with disease activity in rheumatoid arthritis. This study showed that the ratios of ACE1 to ACE2 were higher in RA patients, whereas the counter-regulatory axes Ang II/Ang-1–7 ratios were lower in RA patients (Braz et al. 2021).

Data in the current study revealed that OA causes significant changes in RAS components compared to the healthy control group, showing a significant increment in the levels of ACE1, ACE2, and Ang II, and on the other hand there is a significant decrement in levels of Ang 1–7 and MasR. Such changes were associated with OA disease progression. Intriguingly, combination therapy of HYAL + DIZE showed the most potent effect over single therapies in opposing the variations caused by OA in controlling RAS-related inflammatory components. This combination was able to dampen down the classical RAS axes (ACE1, ACE2, Ang II) and boost the counter-regulatory pathway (Ang 1–7, MasR).

As mentioned earlier, the counter-regulatory axis of RAS has an anti-inflammatory and anti-oxidative stress action (Khajeh Pour et al. 2022). Exogenous administration of Ang 1–7 showed improvement of the inflammatory imbalance in RA rats, an effect that was modulated through the MasR signaling (Khajeh Pour et al. 2022). Another study by Simões e Silva et al. (2013) revealed that the ACE2/Ang-1–7/MasR axis has a great role in modifying processes associated with inflammation including leukocyte influx and fibrogenesis, where those axes have shown a positive role in the regulation of kidney inflammation and fibrosis. The use of DIZE as an ACE2 activator has been shown to be protective in ischemia-induced cardiac pathophysiology decreasing the inflammatory cells in peri-infarct cardiac regions. These beneficial effects associated with DIZE treatment were abolished by an ACE2 inhibitor (Qi et al. 2013). As demonstrated in the current manuscript DIZE (and other treatments) showed an increased ACE2/ACE1 ratio, which corresponds with data of the Ang1-7 and Ang II. This might imply the activation/deactivation of ACE2/ACE1, respectively, in OA upon DIZE treatment. Moreover, DIZE as well as HYAL and their combination improved the functional and structural outcomes of knee OA as seen histologically in parallel to their respective effects on ACE2/ACE1 expression. Bai et al. have also shown a relationship between tissue HYAL and the RAS, where AT1R activation by Ang II increased hyaluronidase activity, an enzyme for hyaluronan degradation in hypertension and myocardial fibrosis male Sprague Dawley rat model (Bai et al. 2016).

Recent studies highlighted the role of oxidative stress in the progression of OA having a definite role in the increment of inflammatory mediators such as TNF-α and IL-6, being accused for the developed chondrocytes inflammation. In addition, the produced ROS during OA results in matrix degradation through the expression of matrix-degrading proteases (Ansari et al. 2020; Peng et al. 2021). In the present study, there were significant elevations in the oxidative stress molecules NOX-4 and MMP-13, in addition to significant elevations in the inflammatory mediators TNF-α and TGF-β in the knee joint homogenate of the OA group. This confirms the fact that oxidative stress molecules and inflammatory mediators are interdependent, both affecting each other (Biswas 2016). Hence, agents having both anti-inflammatory and anti-oxidative stress effects are targets for the treatments of OA, where the combination of HYAL + DIZE in this study was able to fulfill this criterion. Regression analysis in the current study supports the use of this combination showing the synergistic effect of both drugs on TNF-α levels, an effect that might be attributed to their synergistic effect on ACE2, again adding more value to the effect of both drugs on the RAS axis. Other studies have already shown the potent effect of DIZE in decreasing both oxidative stress and inflammation whether it is taken systemically or topically in an endotoxin-induced uveitis mice model, through activation of the ACE2 pathway (Qiu et al. 2014; Zheng et al. 2015). Moreover, intraarticular injection of HYAL is considered a therapeutic option for the treatment of OA as it suppresses inflammatory responses and attenuates oxidative stress and apoptosis in many clinical studies (Altman et al. 2019; Marinho et al. 2021; Gallorini et al. 2022).

## Conclusion

In conclusion, current integrative and molecular data highlight the importance of the combination of both DIZE and HYAL in the treatment of OA through the improvement of behavioral, and biochemical anomalies. Additionally, this study showed the important role of the RAS axis (ACE2/Ang1–7/MasR) in the etiology and immunomodulatory effect of OA. Clinical studies are warranted to ascertain the reprogramming efficacy of this therapeutic combination and to study its side effects and interactions. The long-term effect of such a combination, as well as its side effects, has also to be in focus in future perspectives.

## Data Availability

All data generated or analyzed during this study are included in this published article.
